# How do undergraduates cope with anxiety resulting from active learning practices in introductory biology?

**DOI:** 10.1371/journal.pone.0236558

**Published:** 2020-08-12

**Authors:** Jennifer R. Brigati, Benjamin J. England, Elisabeth E. Schussler

**Affiliations:** 1 Department of Biology, Maryville College, Maryville, Tennessee, United States of America; 2 Department of Ecology and Evolutionary Biology, University of Tennessee–Knoxville, Knoxville, Tennessee, United States of America; University of Texas at Austin, UNITED STATES

## Abstract

Active learning pedagogies decrease failure rates in undergraduate introductory biology courses, but these practices also cause anxiety for some students. Classroom anxiety can impact student learning and has been associated with decreased student retention in the major, but little is known about how students cope with anxiety caused by active learning practices. In this study, we investigated student coping strategies for various types of active learning (clickers, volunteering to answer a question, cold calling, and group work) that were used in 13 introductory Biology courses at a large public university in 2016–2017. A survey asked students to rate their anxiety regarding the four active learning practices and over half of the students explained the coping strategies they used to manage their active learning anxieties. Coping responses from 880 students were sorted into pre-defined categories of coping strategies: problem solving, information seeking, self-reliance, support seeking, accommodation, helplessness, escape, delegation, and isolation. We found that a different category of coping was dominant for each type of active learning. The dominant coping strategies for anxiety associated with clickers, cold calling, and group work were adaptive coping strategies of information seeking, self-reliance, and support-seeking, respectively. The dominant coping strategy for volunteering to answer a question was escape, which is a maladaptive strategy. This study provides a detailed exploration of student self-reported coping in response to active learning practices and suggests several areas that could be foci for future psychosocial interventions to bolster student regulation of their emotions in response to these new classroom practices.

## Introduction

Although student cognition is often the focus of biology education research, there is strong evidence that student emotion plays an equally large role in student classroom success and persistence in academic endeavors [1]. In academic contexts, student emotions are typically called achievement emotions, because they are linked to perceptions of classroom achievement activities or achievement outcomes [2]. The most frequently reported achievement emotions by university students include enjoyment of learning, hope, pride, relief, anger, anxiety, shame, and boredom, with some of these emotions considered pleasant and some considered unpleasant in terms of student experience [3]. The most commonly cited emotion students report is anxiety [3], which is alarming given that anxiety can have a negative impact on student academic trajectories [4–6].

Students can modify achievement emotions through emotion regulation [7, 8]. Indeed, individual response to emotion is complex because it is interrelated with motivation [9, 10], cognition [11], and decision-making [12], to name a few. Given the potential negative impacts of anxiety on student success, and the potential for modification of this emotion through emotion regulation, a better understanding of how students experience and cope with anxiety in the classroom is desirable.

### Anxiety in undergraduate classrooms

**Fig 1:**
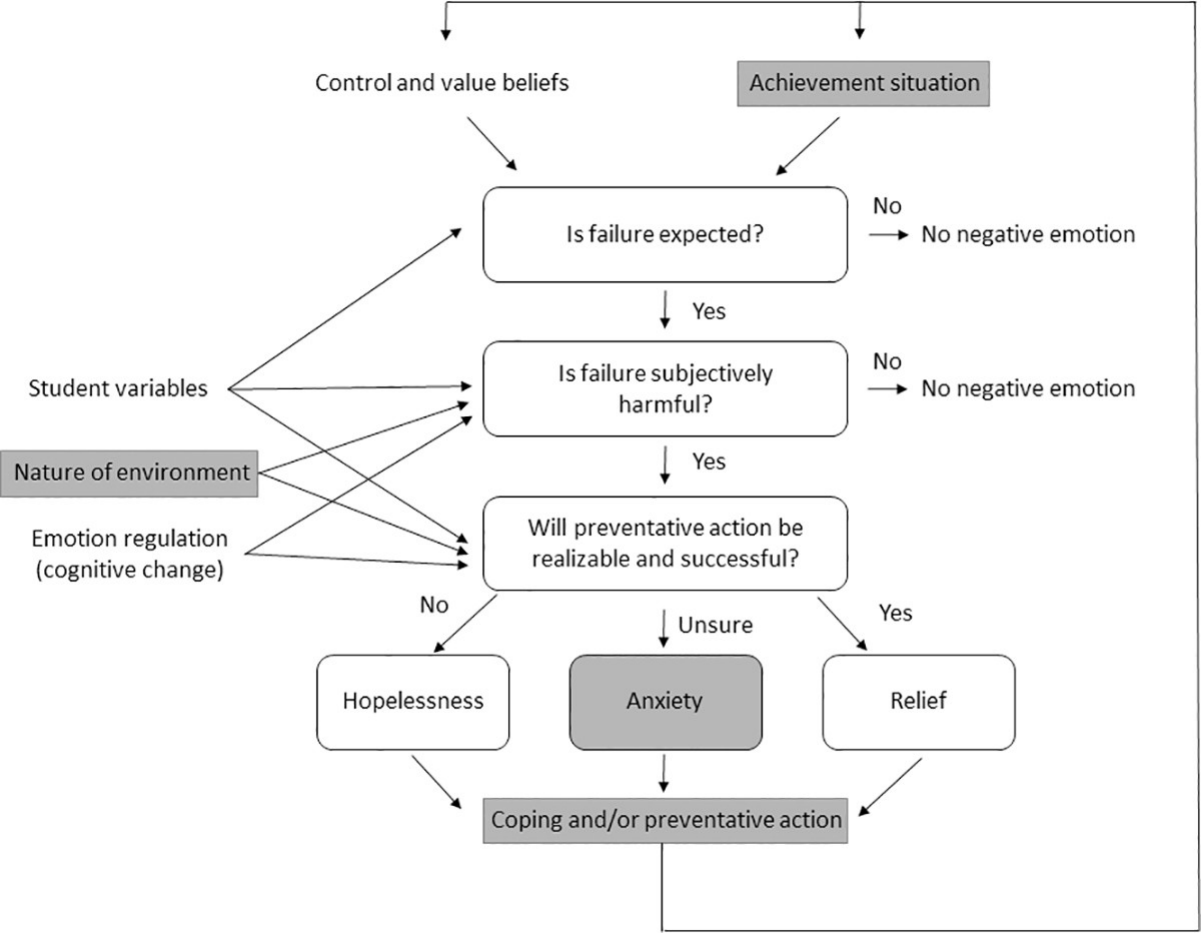
The control-value theory of achievement emotions [2] as well as influences of situational contexts [13, 8] and emotion regulation [7] on student emotions. Items shaded in gray are the focus of this study, in which we examine student coping methods for dealing with anxiety that they associate with various types of active learning in their introductory Biology classrooms.

Pekrun [2] posited the Control-Value Theory of Achievement Emotion as a framework for classroom investigations of student emotion. In this theory, the situational perceived value that students place on the classroom activity or outcome—and the perceived control the students feel they have over the activity or outcomes—are the proximal antecedents to the achievement emotions they feel. In the case of the emotion of anxiety, students perceive that the activity or outcome is valuable to them but are uncertain about the amount of control they have to achieve at their desired level [2]. Students are embedded in social learning environments, however, and these situational contexts also influence their appraisals of control and value as suggested by Social Cognitive Theory [13, 8]. Thus, the generation of emotions is influenced by factors such as the nature of the environment (classroom instruction, university climate) and student variables (emotional predispositions, career goals, previous course experiences), etc. As an example of these impacts, positive nonverbal and verbal instructor communication styles are linked with fewer negative student emotions [14]. Thus, there are multiple factors beyond perceived control and value that interact to influence the emotions that students experience in classrooms, including student personal characteristics, the practices being used in the class, the instructor communication style, and the peer interactions they experience. This is why the same student can experience different emotions in different classes, or even from day to day in the same class. Fig 1 illustrates the interactions between elements of the Control-Value Theory, Social Cognitive Theory, and emotion regulation as they apply to this study.

Anxiety is a prospective emotion; it is a feeling of apprehension that occurs when students appraise an academic demand that they consider important for their success and are uncertain about whether they have the resources (cognitive, motivational, etc.) to achieve what is being asked of them [3, 15]. Anxiety is most often associated with negative impacts on performance [16, 17], but it is classified as an activating emotion, which means that in some cases it can motivate students to improve their performance [18]. For example, some students may feel anxiety about an upcoming exam and use it as a motivator to study, and in so doing, they perform better than another student who feels so much anxiety that they avoid studying and do not do well on the exam.

Testing isn’t the only thing that can cause anxiety in the classroom; low stakes assessments can also cause anxiety [19]. In recent years there has been a movement toward incorporating more active learning into STEM courses [20], with active learning defined as any technique that explicitly asks students to engage with the class material and the instructor, such as answering questions, working problems, or completing peer discussions [21]. As introductory science classrooms shift toward these more student-centered practices, there is a natural increase in daily activities that assess student comprehension (such as clicker, verbal question, and activity responses) as a way to provide formative feedback to students and instructors [22]. This new focus on engaging activities, peer interaction, and formative assessment may alter factors that impact student emotions compared with classrooms where listening and note-taking are the only expectations, as suggested by a recent study by Jacob et al. [23]. For example, students may feel unable to control their success when an instructor calls on them to answer a question in front of the class due to social anxiety, or they may alternatively feel that discussing an idea with peers provides more value to an activity than if they answered the question on their own. In terms of the learning environment, active learning puts a higher priority on social interaction during class time, as well as employs almost daily evaluative feedback. Indeed, studies have found that certain active learning practices, cold calling in particular, are associated with student feelings of anxiety [24–26], suggesting that these new classroom practices are potentially changing the emotional experiences of students in these classes.

Anxiety has been increasingly studied in relation to the types of active learning being used in the classroom because students have variable levels of anxiety depending on the practice being employed. In their investigation of student reactions to classroom active learning practices, Cooper et al [25] found that cold calling was the only active learning practice that students said always increased their anxiety; other types of active learning could sometimes increase or decrease anxiety. England et al [26] found that cold calling and volunteering to answer a question (both of which involve speaking in front of the entire class as well as the instructor) caused higher average student anxiety than working in groups, answering a clicker question, or working on a worksheet (wherein answers may only be shared with small groups of students or the instructor). These anxiety responses are of concern because recent studies have linked overall classroom anxiety with a lack of persistence in majors or institutions [4–6]. It is likely that how students modulate or react to anxiety related to classroom experiences is a significant factor in these persistence outcomes.

### Emotion regulation

Emotion regulation can be defined as the attempts individuals make to control what emotions they feel, the timing of these emotions, and the way they express or experience them [7]. Emotions can be regulated by students by preventing or redirecting their emotional reactions, which can be automatic or involve conscious effort [27]. According to Gross [7], there are five families of emotion regulation strategies; situation selection, situation modification, attention deployment, cognitive change, and response modulation. In academic situations, cognitive change is the most effective strategy for regulation of affective response, and has been shown to improve memory and exam performance [Reviewed in 28, 29]. Cognitive change occurs when an individual modifies their appraisal of a situation [7]; providing just one of many ways that emotion regulation can be linked with Pekrun’s Control Value Theory [2, 8]. For example, a student may re-appraise the perceived value of the activities causing the emotion, or re-adjust the perceived control they have over their success, which will alter the emotional response.

Perceived control [30] has been of interest to researchers as a way to modulate students’ belief in their ability to influence their own success or failure [31]. Perceived control can be viewed as the confidence a person has in the connection between their behavior and a desired outcome, and in academic situations is based on the relationship between a student’s perception of what it takes to do well and their confidence that they have what it takes [32, 33]. Perceived academic control is strongly related to levels of student anxiety, with high control related to low anxiety, and low anxiety related to an intent to persist in a student’s desired major [1].

Negative, stressful emotions can be regulated by employing coping strategies, which are behaviors, cognitions, or perceptions enacted by an individual to reduce or resolve feelings of anxiety or stress [34–36]. Coping and emotion regulation are similar in that both aim to regulate emotion, both may involve purposeful efforts, and both can change over time, however, coping is distinct from emotion regulation in that it occurs only in response to negative emotional states and tends to be an internally-focused process [37]. Coping is employed when an individual believes that the demand of the task is greater than the personal resources they have to achieve the desired outcome [15]. Thus, an active learning task presented in class may cause some students anxiety when they feel they do not have the resources to answer the question appropriately, while other students may feel no anxiety from the same task because they feel they have the resources to address the question. Given the known association of anxiety with different types of active learning in university classrooms, it seems necessary to better understand the coping strategies used by undergraduates when dealing with these anxieties.

### Coping strategies

Generalizations about coping strategies are difficult because researchers do not agree on categorization of the approaches, coping results are often contextually-bound, and individuals can vary in their coping responses. Most researchers agree that coping strategies are difficult to classify into uniformly “good” or “bad” strategies. The effectiveness of the strategy is based on the anxiety outcome, and what works for one individual may not be the same as what works for someone else. Coping strategies that effectively manage or reduce anxiety are generally considered adaptive, and those which do not are maladaptive [38–40]. Individuals who use more *active* coping strategies, where they enact methods to manage the environment to reduce their anxiety (related to perceived control), are typically better at coping with stress [41]. In studies of college students, for example, active coping is a positive strategy in terms of adjustment and persistence [42, 43]. The concept of “approach” versus “avoidance” is based on the idea that individuals either actively use strategies to engage with and reduce the stressor, or enact strategies to withdraw from or help distract them from the stressor [44–46]. Although either can be an adaptive strategy (avoidance is an effective strategy for uncontrollable situations, for example), long-term and repeated use of avoidance strategies is widely considered maladaptive [47].

Skinner et al. [35], reviewed over 100 coping classification schemes and proposed a structural organization within which to organize coping strategies. The classification structure we used in this study was based on their 12 higher order action types of coping that are classified under three overarching concerns: competence, relatedness, and autonomy [48–50]. These three concerns are the psychological needs identified in self-determination theory [51]. In Skinner’s structural organization of coping, each of the three concerns has two approach strategies and two avoidance strategies. The two approach and avoidance strategies are delineated by appraisals of challenges / threats to the self and challenges / threats to the context [18]. Thus, the coping category structure has two challenges and two threats to competence, two challenges and two threats to relatedness, and two challenges and two threats to autonomy. The resulting 12 coping strategies can also be sorted into adaptive, maladaptive, and either adaptive or maladaptive categories as informed by Skinner et al. [35] and Henry et al. [39].

As an example, the concern of relatedness has to do with peer and self interactions. The interactions that are considered adaptive are self-reliant approaches and seeking support. These include relying on yourself to modify your emotional reactions or going to peers or even a religious faith to find support (approach strategies). The interactions that are considered maladaptive are delegation and isolation (avoidance strategies). Delegation is feeling sorry for oneself or depending on others to do the anxiety-provoking task, and isolation is to remove oneself from peer interaction. An overview of the 12 coping categories we used from Skinner et al. [35] is provided in Table 1.

**Table 1:** Categories (12) of coping responses, with brief descriptions, identified by Skinner et al. [35] and further elaborated by Henry et al. [39].

Response to Challenges/ Threats Toward:	Adaptive Coping Responses	Maladaptive Coping Responses
**Competence**	**1. Problem solving** • strategizing • instrumental action • planning **2. Information seeking** • reading • observation • asking others	**3. Helplessness** • confusion • cognitive interference • cognitive exhaustion **4. Escape** • avoidance • denial • wishful thinking
**Relatedness**	**5. Self-reliance** • self-regulation • emotional expression • emotional approach **6. Support seeking** • contact seeking • comfort seeking • spiritual support	**7. Delegation** • maladaptive help-seeking • whining/complaining • self-pity **8. Isolation** • social withdrawal • concealment • avoiding others
**Autonomy**	**9. Accommodation** • distraction or minimization • cognitive restructuring • acceptance ***10*. *Negotiation*** • *bargaining* • *persuasion* • *priority setting*	**11. Submission** • rumination • rigid perseverance • intrusive thoughts **12. Opposition** • other-blame • projection • aggression

Coping strategies can be generally considered adaptive, maladaptive, or either (italicized; depends on the stressor). These are further subdivided by the responses to challenges or threats to individuals’ competence, relatedness, or autonomy.

### The current study

Previous research at this university [4, 26] identified that students in Introductory Biology courses experienced different levels of anxiety towards different active learning practices such as clicker use, working in groups, volunteering to answer a question, and cold calling, and that anxiety in general can impact persistence in the major. Anxiety levels differed among students and among different demographic subsets of students in the classes, with women and students who took fewer AP courses in high school having higher anxiety than men and those who took more AP courses [4]. Average student anxiety levels also differed among different introductory Biology classes, suggesting instructor practices may also differentially influence student emotional reactions. Our research to date, however, has not explored how students respond to the anxieties they feel when active learning is used in their classes. Understanding the coping strategies students are using is important to evaluate the potential success of these strategies, and direct further research toward interventions that may help students better regulate their anxiety.

The goal of this research was to categorize and summarize the coping strategies students used in response to anxiety they associated with different active learning practices. The investigation of coping strategies specific to typical active learning practices in introductory Biology classrooms is in its infancy; thus, we took a qualitative exploratory approach to inform future research on this topic. We asked students in multiple introductory Biology classes about their anxiety levels regarding four active learning practices and the coping strategies they deployed when those practices were used. We categorized these coping strategies and asked two research questions:

1. What types of coping strategies are students using in response to active learning practices?
2. Do student coping strategies vary by the type of active learning practice used?

## Methods

### Ethics statement

All procedures for this study were approved by the University’s Institutional Review Board prior to the start of the research (IRB-16-03181-XP). Electronic informed consent was obtained from participants; at the beginning of the electronic survey, consent information approved by the institutional review board was presented, and students then indicated via a yes or no click of radio buttons whether they were willing to have their data used as part of the project.

### Courses and instructors

Data were collected during the 2016–2017 academic school year from students enrolled in several majors’ introductory Biology lecture classes at a large Southeastern public research university. An online survey was used to collect student self-reported anxiety level toward several classroom active learning practices, coping strategies enacted in response to each active learning practice, lecture section, and student demographics. The four active learning practices probed were clicker questions, volunteering to answer a question, cold calling, and group work. The introductory Biology sequence at this university includes one 3-credit Organismal and Ecological Biology (OEB) course and one 3-credit Cellular and Molecular Biology (CMB) course. These courses are taken by mostly freshman and sophomore students seeking a science or pre-professional degree. The year of this study, enrollment in each lecture section ranged from 49–230 students. Thirteen lecture classes were surveyed for this study (encompassing 6 lecture classes in the fall (3 OEB and 3 CMB) and 7 lecture classes in the spring (3 OEB and 4 CMB).

The OEB and CMB courses were revised in 2014–2015 to incorporate the main tenets of *Vision and Change in Undergraduate Biology Education* [52]. This included integrating the 5 core concepts for biological literacy and the 6 core competencies and disciplinary practices identified in *Vision and Change* into the OEB and CMB curriculum, and shifting toward student-centered learning by incorporating active learning and formative assessment into each class. While instructors were not required to use any particular type of active learning, most began using clickers (response systems that allow all students in the class to respond electronically to questions asked in lecture in real time) and worksheets (short written assignments or worksheets completed in groups of 2–10 students), and asked verbal questions then waited for a response by a student volunteer or called on a student by name for a response (“cold call”). Not all instructors used all of the active learning practices, however. The instructors of the courses all held doctorates and were part of a programmatic community who met to talk about implementing active learning in the introductory courses.

The active learning practices used in these classes were initially confirmed by studies investigating how active learning was enacted and changed over time in these two courses as part of a curriculum reform project [53, 54]. In these studies, classroom events were classified as active learning if the instructor explicitly asked the students to engage with the material in some manner (i.e., answer a clicker question, discuss a concept, draw a phylogenetic tree), while lecture, video, logistical questions posed by the instructor, and student questions of the instructor were not considered active learning [53]. These studies found that active learning in these classes ranged from 13% to 53% of class time, depending on the instructor, and averaged 39% of class time across the program in 2014–2015 [53]. The authors of the current study have investigated these introductory courses together since 2015, including regular instructor observations. This research team confirmed previous work [53] that active learning practices in the 13 lecture classes took three main forms: clicker questions (or other electronic class response system), verbal questions (with volunteer responses or cold calling), and written activities.

In 2016–2017, since the author team could not observe all sessions of all of the courses, they had instructors fill out self-report forms at the end of each semester which asked how often they used each of the four active learning practices that semester (never, rarely, occasionally, often, every class) and how often group discussion was used with each practice. All instructors that year asked for student volunteers to answer questions, and all used written group work; however, the use of written group work was often sporadic. Cold calling and clickers were used in 10 of 13 lecture classes. Most instructors allowed group discussion prior to students answering questions in the class.

### Data collection

Data from undergraduate students in these classes were collected with an online survey (via the Qualtrics software) sent via e-mail. The survey was sent to students from instructors who were told about the study and confirmed their willingness to pass on the participation information to their students. These instructors included thirteen of the fourteen introductory lecture classes (OEB or CMB) that academic year. Surveys were disseminated at approximately 4 weeks into each semester (fall and spring) and remained open for 10 days. The e-mail that was sent to students had a brief description of the project and survey, along with information about the incentive and closing date. When students clicked on the survey, consent information that was approved by the institutional review board was presented to obtain informed consent. Students then indicated via a yes or no click of radio buttons whether they were willing to have their data used as part of the project (they could respond and receive the incentive without having their data used) and whether they were over the age of 18.

The survey included four single-item questions that used a 7-point Likert scale (1 = no anxiety, 7 = high anxiety) to probe student anxiety toward four active learning practices: clickers, volunteering to answer a question, cold calling, and group work [26]. There was also an option students could click indicating that this active learning practice was “not used in my class.” The anxiety question was specifically worded: “Rate each of the following active learning practices based on how much ANXIETY they cause you to feel during class this semester. Use the following scale: 1 = no anxiety, 4 = moderate anxiety, 7 = high anxiety. Please note: "clickers/clicker questions" includes questions asked via Learning Catalytics.” The four items that followed were:

You are asked to respond to a clicker question.

You are asked to volunteer to answer a question the instructor has posed.

You are called on by name to answer a question the instructor has posed.

You are asked to work with others in class to answer questions or complete an in-class activity.

Single-item indicators were used because these questions were a part of a larger survey that included numerous validated scales for various types of classroom anxiety, and we were concerned that adding additional length would decrease the response rate. Although single-item measures are not best practice for measuring a latent construct, our intent was not to provide an exact measure of anxiety; instead the intent was to get students thinking about their relative anxiety levels toward each of the active learning practices, as a precursor to asking them about how they reduced any anxiety they felt. These single-item anxiety ratings were not used for any statistical analysis other than basic mean and standard deviation calculations to assess relative anxiety levels of the sample of students toward the various active learning practices.

After indicating their relative anxiety toward each active learning practice, students were asked to respond to four open-ended questions about how they coped with their anxiety for each practice. Specifically, students were asked, “When you are [asked to answer a clicker question; asked to volunteer to answer a question; called on by name to answer a question; asked to work with others in class to complete an activity or answer questions], what do you do to reduce the anxiety you feel?” Participants were instructed to leave the response blank if the active learning practice did not cause them anxiety. Thus, not all students were expected to respond to this question.

Students also provided information about their year in school (drop down menu of freshman through super-senior), gender (drop down menu of: female, male, prefer not to answer, open response), ethnicity (open response question), and instructor / lecture section (drop down menu of instructors). The survey also included scales for general class anxiety, test anxiety, communication anxiety, and other items that were not used in this study.

The total time for survey completion averaged 5–10 minutes. Instructors each offered students an incentive to complete the survey in the form of 1 bonus point. The total points in each class were 1,000, making the incentive a minor part of a student’s final grade.

### Data analysis

Prior to data cleaning, there were 841 students in the fall and 768 students in the spring who responded to the survey. Survey responses from students under the age of 18 and those who did not consent to the use of their survey data for research purposes were removed from the data set. We further removed participants who did not answer the four questions related to anxiety about each active learning practice. This represented the sample of students from which we examined coping responses (anxiety participants); However, some of these students provided no coping answers because they may not have felt anxiety about the active learning practices. We thus created a separate data set for each active learning practice that included only students who had responded to any of the coping questions (coping participants). Some students provided one coping answer regarding one active learning practice and some provided up to four responses, one for each active learning practice.

#### Anxiety levels

For the participants who answered all the anxiety questions, we examined the distribution of anxiety ratings for each active learning practice, as well as the average anxiety (and standard deviation) for each practice. We also tallied the number of students who indicated that an active learning practice was not used in their class. This provided information about the relative anxiety levels that students in this sample felt toward the active learning practices. We then compared these numbers with the same tallies and calculations for the subset of students who provided coping responses (coping participants).

#### Coping categorization

This portion of the study only used the coping participant responses to each active learning practice. Rather than inductively defining categories of coping strategies from the student open responses, we used the 12 coping categories identified and described by Skinner et al. [35] shown in Table 1.

Two of the authors (JB and ES) conducted the qualitative analysis. Both were immersed in studies of teaching practices and student responses to those teaching practices in the introductory Biology classes that academic year. They had also worked as a team on previous qualitative coding projects. To code student open-ended responses using the Skinner categories, the researchers decided to follow a deductive content analysis approach [55]. The researchers each read the Skinner paper and discussed their understanding of the 12 coping strategies and their suitability for coding the student responses. They took notes on the categories to create an initial codebook and decided to each individually read through the student responses and refine the codebook further using typical student responses. While individually reading the student responses, they made notes about student responses that seemed to fall into each category or ones that they struggled to categorize. After this process, the researchers met and discussed how they had defined and used each code and compared examples of student responses that they believed fit into each category. The researchers discussed categories and student responses where they had differing opinions until they reached a mutual understanding of the categories. The researchers found that neither had identified responses that fell into the coping categories of *negotiation*, *submission*, and *opposition*, so these categories were dropped from the study. All of these codes fell into the autonomy portion of Skinner’s framework. This process resulted in a codebook with nine codes (plus a “no code” category) with category descriptions that related specifically to how students were coping to active learning practices (brief descriptions in Table 2 and codebook examples in S1 Table).

**Table 2:** Categories (9) of coping responses that students reported when dealing with anxiety associated with active learning practices, and examples of each category from the researcher’s codebook.

Response to Challenges/ Threats to:	Adaptive Coping Responses	Maladaptive Coping Responses
**Competence**	**1. Problem solving** • use logic or focus • work through the problem • repeat the question in my own words **2. Information seeking** • use your notes or book • prepare for class • ask a classmate or TA	**3. Helplessness** • freeze or panic • randomly guess • just hope for the best **4. Escape** • eye contact • don’t answer/lose attendance points • don’t raise my hand
**Relatedness**	**5. Self-reliance** • breathe, relax • use positive self-talk • be confident/ psych yourself up **6. Support seeking** • work with people you know • give answer from the group • use social skills to make group work more comfortable	**7. Delegation** • just listen • talk as little as possible • let someone else start the discussion **8. Isolation** • work on my own
**Autonomy**	**9. Accommodation** • just answer • just do the best I can • answer quickly to get it over with	N/A

The researchers then decided on a strategy for coding all of the student responses. They defined a coding unit as an entire student response regarding how they reduced anxiety for one active learning practice. The researchers assigned as many codes to the student response as they thought captured the coping ideas expressed by the student. This was because many students expressed two different ideas in one response, such as one student who said, “Take a deep breath and talk through the solution with those around me,” which expressed both self-reliance (emotion regulation via breath control) and problem-solving (gathering information from other students). This meant that each coder may have given different numbers of codes per student response.

Using the codebook for the nine coping categories developed through initial analysis (Table 2, S1 Table), the researchers proceeded to independently code the first 100 responses of each active learning strategy. They then met to compare their coding, resolve differences, and continue to refine the codebook based on their disagreements. After this process, they felt confident that they could reliably code the rest of the data. They then independently coded the rest of the data set, after which the two researchers met again and resolved any differences in coding via discussion and reconciliation to complete agreement. Throughout the process, iterative edits were made to the codebook. Before reconciliation, the researchers agreed on 81% of the codes they assigned to student responses. Interrater reliability was calculated for each active learning practice and all data combined using Cohen’s kappa. Kappa is considered a better reliability measure than percent agreement between raters because it accounts for the possibility that chance agreements may have occurred [56]. Kappa values between 0.61 and 0.80 are considered substantial agreement, while those from 0.81 to 1.00 are considered almost perfect agreement [57]. Because coders could assign more than one code per coding unit, there was not always a one to one comparison of codes that could be made. In these cases, one of the codes was paired with “no code” as the comparable category.

After all the codes were assigned, the researchers compiled the results for each active learning practice. The number of codes for each coping category was tallied for each active learning practice. Because all strategies mentioned by the students were provided a code (even within the same response), the number of coping strategies for each active learning practice exceeded the number of student responses. The number of coping strategies mentioned for each active learning practice was organized as a distribution and converted into a percent by dividing the number of times the code was identified by the total number of student responses for that active learning practice. This represented the percent of students who used strategies related to each coping category for each active learning practice. We also examined the responses of individual students to tally how often a student used the same coping strategy for different active learning methods.

## Results

### Participants

There were 841 initial student survey responses in the fall and 768 initial student survey responses in the spring. After removing responses for students who did not consent to the study, those who were under the age of 18, and those who did not complete the anxiety questions, there were 1,425 student responses (750 in the fall and 675 in the spring). Of these students, 70 were present in both the fall and spring data set because they took two of the introductory biology courses over the academic year. A review of their coping responses found that they were rarely identical from one semester to the next, and these students were retained in the data set. The percent of student respondents for each lecture class ranged from a low of 31% of the students in one lecture class up to 90% of the students in another lecture class. There was no clear relationship between the amount of self-reported active learning use by an instructor and the response rate of students in their class. One possible explanation for the variability in response rates is that the survey (and associated extra credit) may have been promoted in class by some instructors, and reminder emails may have been sent by some instructors, while others simply forwarded a single survey email to their students.

The number of students who provided both anxiety ratings and coping responses to at least one active learning practice was 880. Of these, 478 were students from the fall semester, and 402 were from the spring semester. These 880 students provided a total of 1,748 unique responses to the questions about coping. Demographic information for the participants who completed all anxiety questions and the coping questions is shown in Table 3.

**Table 3:** Demographic information provided by participants who completed both the anxiety questions and at least one coping response (n = 880).

Year in School	Percent of respondents
Freshman	49%
Sophomore	35%
Junior	12%
Senior or above	5%
**Gender**	
Female	72%
Male	27%
Non-binary or open response	<1%
**Ethnicity**	
White	78%
Non-white*	21%
No response	<1%

*The non-white category was determined by open responses in which students indicated a race or ethnicity that was not white or Caucasian. Responses in this category included Asian (about 20% of non-white responses), Hispanic / Latinx (about 12% of this category), African American / Black (31%), Mixed race (14%), and many other identities.

### Student anxiety levels vary by active learning practice

For the anxiety response data set, it appeared that more students reported the highest level (7) of anxiety for volunteering to answer a question (mean = 4.04) and having an instructor call on you by name (mean = 4.82) than any other anxiety level. When asked about anxiety associated with answering a clicker question or performing group work, the most common responses were in the moderate anxiety range (3–5; clicker mean = 2.67; group work mean = 2.65) (Table 4). The number of students reporting that cold calling was not used in their class appeared to be higher than other practices, despite the high anxiety students reported about this practice. The average anxiety level students reported for each active learning practice appeared to be higher for the subset of students who provided coping strategies, which was expected given the prompt to only provide a response if the student felt anxiety for that practice (Table 4).

**Table 4:** Average and distribution of self-reported anxiety of students in response to various types of active learning.

	Active Learning Practice							
	CQ		V		C		GW	
	All	+ Coping	All	+ Coping	All	+ Coping	All	+ Coping
Number of responses	1087	354	1297	534	1149	532	1396	328
Average anxiety	2.67	3.83	4.04	4.87	4.82	5.47	2.65	4.05
Standard deviation	1.71	1.56	1.97	1.67	2.05	1.59	1.74	1.65
Anxiety distribution								
1	389 (35.8%)	18 (5.1%)	175 (13.5%)	12 (2.2%)	117 (10.2%)	6 (1.1%)	498 (35.7%)	13 (4.0%)
2	208 (19.1%)	53 (15.0%)	165 (12.7%)	39 (7.3%)	90 (7.8%)	23 (4.3%)	310 (22.2%)	58 (17.7%)
3	160 (14.7%)	81 (22.9%)	194 (15.0%)	64 (12.0%)	103 (9.0%)	39 (7.3%)	172 (12.3%)	53 (16.2%)
4	156 (14.4%)	77 (21.8%)	209 (16.1%)	86 (16.1%)	157 (13.7%)	71 (13.3%)	189 (13.5%)	75 (22.9%)
5	92 (8.5%)	57 (16.1%)	206 (15.9%)	119 (22.3%)	144 (12.5%)	68 (12.8%)	107 (7.7%)	58 (17.7%)
6	44 (4.0%)	29 (8.2%)	145 (11.2%)	74 (13.9%)	186 (16.2%)	107 (20.1%)	66 (4.7%)	36 (11.0%)
7	38 (3.5%)	23 (6.5%)	203 (15.7%)	120 (22.5%)	352 (30.6%)	185 (34.8%)	54 (3.9%)	31 (9.5%)
Not used in my class	339	See below	128	See below	276	See below	27	See below
Not used in my class, but still provided a coping strategy	16		19		32		4	
No anxiety reported, but still provided a coping strategy	0		1		1		0	

CQ = answering a clicker question; V = instructor asks for a volunteer; C = instructor calls on student by name; GW = working in groups.

“All” represents the data set with students who answered the anxiety questions, with calculations of anxiety excluding those students who indicated that the practice was not used in their class. The phrase “+ coping” represents the data set of students who provided anxiety and coping responses. For both groups, average anxiety, standard deviation of anxiety, and the distribution of responses along the Likert scale (1 = no anxiety, 4 = moderate anxiety, 7 = high anxiety) are provided. Also reported is the count of students who indicated an active learning practice was not used in their class (as well as those who still provided a coping strategy despite that) as well as students who did not report anxiety but still provided a coping strategy.

### Student coping strategies vary by active learning practice

The coding process resulted in high interrater reliability. Kappa for the clickers portion of the data set was 0.792 (SE = 0.022). Coding of the data related to volunteering to answer a question had a kappa of 0.837 (SE = 0.016), while cold calling had a kappa of 0.756 (SE = 0.018). Coding for the group work data set had a kappa of 0.723 (SE = 0.025). Finally, coding for the entire data set had a kappa of 0.805 (SE = 0.009).

The number of students reporting a coping strategy appeared to vary by active learning method, with fewer coping strategies being provided for the two methods that students appeared to associate with less anxiety (clickers, group work). Of the 1,748 student coping responses given by the 880 students, 354 were responses for clickers, 534 were responses for volunteering to answer a question, 532 were responses for cold calling, and 328 were responses for group work. Of the 2,089 potential participants in the classes, 17% of students provided coping responses regarding clicker use, 26% about volunteering to answer a question, 26% about cold calling, and 16% about group work. There were only 57 students (3% of potential sample) who gave a coping response for all four active learning practices. There were 198 students who provided coping responses for three active learning practices, 304 for two practices, and 321 for one practice only. All student coping responses have been provided in S1 Appendix.

Individual student responses could be assigned multiple coping codes if they expressed more than one coping strategy. Of the 1,748 student coping responses, 1,417 expressed ideas coded into a single category (e.g., their response only conveyed ideas related to “information seeking”), while 316 student responses were coded into 2 categories, 11 student responses were coded into 3 categories, and 4 were coded into 4 categories. The percentage of responses coded into more than one category were fairly even across the four active learning practices (15% - 22%). A breakdown of binning per category is provided in Table 5.

**Table 5:** Number of student coping responses (strategies) organized by how many categories into which they were placed.

	Number (percentage) of responses per active learning practice			
Number of coping categories into which responses were placed	Answering a clicker question (354 responses)	Instructor asks for a volunteer (534 responses)	Instructor calls on student by name (532 responses)	Working in groups (328 responses)
1	287 (81.1%)	434 (81.3%)	416 (78.2%)	280 (85.4%)
2	66 (18.6%)	91 (17.0%)	113 (21.2%)	46 (14.0%)
3	0 (0%)	8 (1.5%)	2 (0.4%)	1 (0.3%)
4	1 (0.3%)	1 (0.2%)	1 (0.2%)	1 (0.3%)

Student coping strategies were binned into appropriate categories based on Skinner et al. [35] and Henry et al. [39]—there were ultimately nine coping strategies (categories) students reported. Some students reported more than one strategy for each practice; thus, there were some responses coded into 2, 3, or even 4 coping strategies. The number of categories into which student responses were placed is listed above. For example, there was one clicker-related student coping response (out of 354) that was placed into 4 separate coping categories.

The distribution of the coping strategies used by students for each active learning practice is shown in Table 6. These distributions generally appeared different for each active learning practice, although some coping strategies were common for multiple types of active learning. Students used strategies sorted into 8 of the 9 coping categories for anxiety associated with being asked to answer a clicker question, but did not use strategies categorized as delegation. Students also reported using strategies sorted into 8 of the 9 coping categories when being asked to volunteer to answer a question or being called on directly to answer a question, but did not use strategies categorized as isolation. Students used coping methods in all 9 categories when dealing with anxiety associated with group work. Examples of student responses, and the coping categories they were sorted into, can be found in S1 Table.

**Table 6:** Distribution of coping strategies used by students in response to anxiety associated with different active learning practices.

	Number (percentage) of responses placed into each coping category for each active learning practice			
Coping categories	Answering a clicker question (354 responses)	Instructor asks for a volunteer (534 responses)	Instructor calls on student by name (532 responses)	Working in groups (328 responses)
Problem solving	86 (24.3%)	99 (18.5%)	137 (25.8%)	25 (7.6%)
Information seeking	194 (54.8%)	63 (11.8%)	49 (9.2%)	42 (12.8%)
Helplessness	17 (4.8%)	23 (4.3%)	60 (11.3%)	4 (1.2%)
Escape	1 (0.3%)	255 (47.8%)	31 (5.8%)	9 (2.7%)
Self-reliance	66 (18.6%)	106 (19.9%)	222 (41.8%)	38 (11.6%)
Support seeking	4 (1.1%)	3 (0.6%)	5 (0.9%)	174 (53.0%)
Delegation	0 (0%)	33 (6.2%)	1 (0.2%)	23 (7.0%)
Isolation	1 (0.3%)	0 (0%)	0 (0%)	31 (9.5%)
Accommodation	54 (15.3%)	62 (11.6%)	147 (27.6%)	33 (10.1%)

Student coping strategies were binned into appropriate categories based on Skinner et al. [35] and Henry et al. [39]—there were ultimately nine coping strategies (categories) students reported. The number of responses that were binned into each category is reported above. Note that some students reported more than one strategy; thus, there were some responses coded into 2, 3, or even 4 coping strategies.

The categories of coping used by students varied among the active learning practices with a different coping category dominating each different practice (Fig 1). When students reported feeling anxiety associated with a clicker question, they most often coped with an information-seeking behavior in which they found resources (including peers) to help them respond to the active learning prompt. Typical responses from students sorted into this category included, “Ask people around me to help,” “I try to look through my notes to find an answer to the question if I do not already know the answer,” and “I usually study more so I know the answers.” The second-most common way that students coped with anxiety associated with a clicker questions was with problem-solving which involved the self-use of logic. Typical responses in this category included, “I just thoroughly read the question and answer options before responding,” “I make sure I have my clicker ready with the correct channel,” and “I try to eliminate answers I know to be false.” All of the above coping examples were adaptive, indicating the students were doing something that reduced their anxiety in a healthy or non-harmful manner.

**Fig 2:**
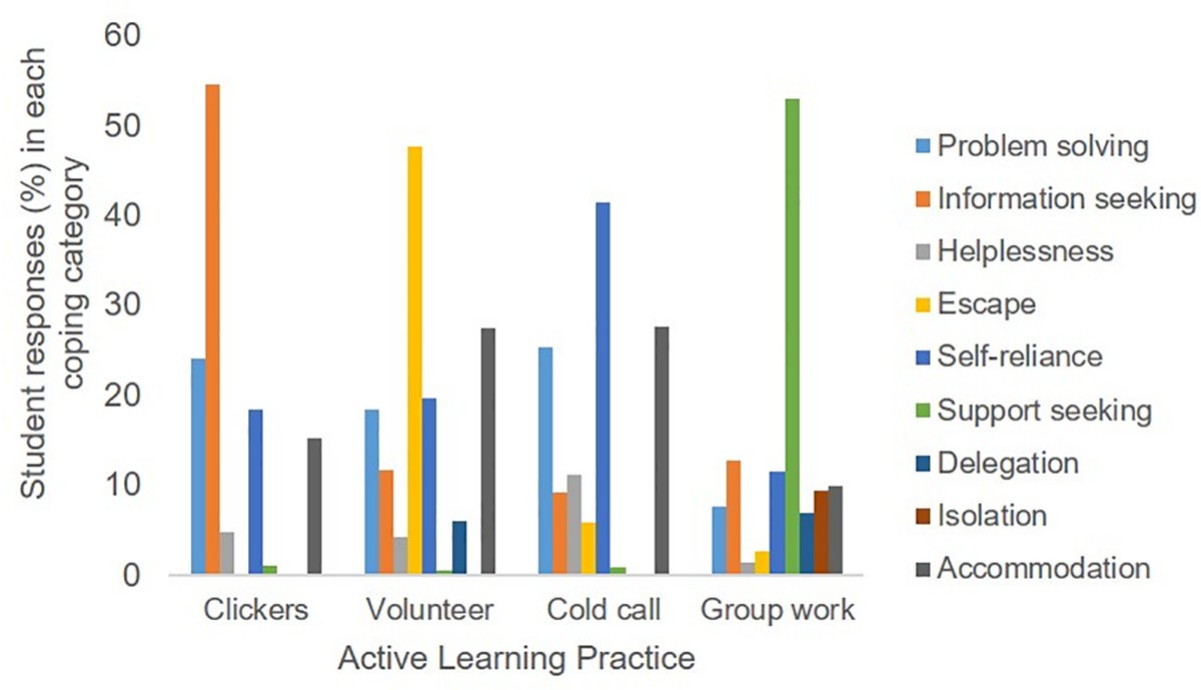
Percent of student responses binned into each of the coping categories for each active learning practice as reported by students (n = 880) enrolled in introductory Biology courses.

When the anxiety was associated with an instructor asking a question and then waiting for a volunteer to answer, the most common type of coping used was escape in which students avoided the active learning situation. Typical responses in this category included, “I just do not raise my hand to answer questions,” “I try not to make eye contact with the professor so he will not call on me,” and “I duck so I don't get picked.” These coping strategies are maladaptive, because the students ease their anxiety but miss out on the opportunity to get feedback from the instructor on their answer. The second-most common way that students coped with anxiety associated with an instructor asking a question and then waiting for a volunteer to answer was self-reliance (an adaptive coping mechanism), followed very closely by problem solving (also adaptive). Self-reliance is internal regulation and self-talk that helps students approach the situation. Typical responses in the self-reliance category included “I take deep breathes,” and “I remember that there is no shame in answering incorrectly,” and in the problem-solving category included, “I prepare my answer in my head beforehand,” and “I try to recall or think of the best possible answer.”

To cope with anxiety students associated with cold calling, they most often turned to self-reliance. Once again, “I take a deep breath,” was a common response in this category, along with positive self talk such as, “I remind myself that if I get it wrong it isn't that bad!” and various types of fidgeting including, “I spin a ring on my finger.” These are all adaptive forms of coping. The second-most common way that students coped with anxiety associated with cold calling was accommodation, followed very closely by problem solving. Accommodation was a response in which students addressed the active learning prompt, but did so quickly and with little reflection. Typical responses in the accommodation category included, “Answer as quickly as possible,” and “Just answer the question,” and in the problem solving category included, “I do my best to think through the question carefully to give a good response,” and “I take a moment to compose my response before speaking.” The strategies falling into the accommodation category were maladaptive, in that the students’ focus was on getting the anxiety-inducing activity over as quickly as possible, rather than using it as a learning opportunity.

When group work was associated with student reports of anxiety, they most often used support-seeking behaviors to cope. These adaptive support-seeking behaviors focused on working with friends and creating a supportive group by being friendly or social, while not necessarily seeking information from them. Typical responses included, “I try to be as friendly as possible to who I am sitting next to,” “I try to sit with my friends,” and “Make a joke, find a commonality with my neighbors/group to ease anxious tensions.” Coping strategies that did not fall into the support-seeking category were fairly evenly distributed among 6 of the 8 remaining categories, with very few students relying on strategies that fell into the hopelessness or escape categories. It is worth noting that about 9% of the students providing coping strategies dealt with group work by isolating themselves and not actually working with a group. Although this is a maladaptive strategy, since the students missed out on the benefits of group work, several noted that there wasn’t a grade penalty for not working in a group so it can be inferred that they didn’t perceive this strategy as having any negative consequences.

Supporting the observation of students generally using different coping strategies for each active learning practice, we examined each student’s coping responses to see how often they employed the same coping strategy regardless of active learning practice. For the 304 students who provided coping responses to two different active learning practices, 114 of those responses shared the same coping code. Of these, self-reliance was the most commonly shared code (N = 48), followed by problem-solving (N = 24) and information seeking (N = 23). For the 198 students who provided three coping responses, only 26 used the same coping strategy for each active learning practice. In this case self-reliance was once again common (N = 8), but followed by problem-solving (N = 7) and accommodation (N = 7). For the 57 students who provided coping responses for all four active learning strategies, only four used the same coping strategy for each; two used self-reliance, and two used information seeking for all forms of active learning.

## Discussion

This research re-confirmed that students experience anxiety when active learning practices are used in the classroom [24, 25, 26]. However, active learning techniques improve student performance in STEM courses [22], so instructors are advised to use these practices despite their potential association with anxiety. Anxiety is not necessarily bad, as the strategies students use for coping with anxiety can have positive impacts on their performance (for example, if they respond by reading the textbook before attending class, they are more prepared for class). However, high anxiety has been associated with negative impacts on student persistence in the major [4, 26], so this study was an exploration into the coping strategies students used for dealing with anxiety associated with different types of active learning.

Students in the introductory Biology courses OEB and CMB were most commonly asked to complete active learning tasks that fell into four categories: answering clicker questions, being asked to volunteer to answer a question, being called upon directly to answer a question (“cold calling”), and completing group work (usually in written form). For the sample examined in this study, there appeared to be differences in the distribution of coping strategies for each of these four practices, and there was a different coping strategy used most commonly for each practice. Even individual students rarely used the same coping strategy for different active learning practices. Harley et al.’s [8] integrated model of emotion regulation in achievement situations may help guide the explanation for these findings because different active learning practices may vary in situation factors, attention factors, as well as appraisal assessments, and each may cause students to employ different emotion regulation strategies (e.g. evaluation factors are typically modified by situation selection or situation modification). For example, a group worksheet is highly social but low evaluative, while a clicker question (in the context of our sample) is moderately social and moderately evaluative. The emotion regulation strategies (which include coping) a student would use for each would thus be expected to vary according to the model.

### Students use mostly adaptive coping strategies

The most common coping responses to anxiety associated with clicker questions, cold calling, and group work appeared to be adaptive coping strategies (information seeking, self-reliance and support-seeking), while the most common response to an instructor request for a volunteer to answer a question appeared to be a maladaptive coping strategy (escape) [35, 39]. This is intriguing because volunteering to answer a question is the only active learning practice where a student gets to choose whether they respond. It is possible that the option of not responding—but perhaps feeling like they should respond—causes internal conflict in students that generates ambivalence and maladaptive coping, whereas having to answer a query (such as for cold calling)—even if it causes more anxiety—results in students employing more adaptive strategies.

Overall, the most common adaptive strategies used by students in these classes appeared to be information seeking, problem-solving, self-reliance, and support seeking, although the latter seemed to be specific to active learning where group work was involved. Indeed, when individual students used the same strategy repeatedly regardless of active learning practice, it was the strategies of information seeking, problem-solving, and self-reliance that were the most common practices. Although the actual efficacy of these strategies is not known, these may be a good place to start when building potential interventions for classroom anxiety. Students could be provided with example strategies that peers in their classes use that fall into these categories, like reading the questions carefully and writing out answers prior to responding (problem-solving), using notes to prepare responses and using peers as sources of information (information seeking), or taking deep breaths, being confident, and practicing positive self-talk (self-reliance). These may be provided as a practical list of strategies that students can try out in their classes to see which works best for them for each active learning practice.

Adaptive and maladaptive categories, however, do not indicate that the coping strategy is more or less likely to result in a negative academic outcome. Here, the adaptive strategies are responses to perceived challenges that the student feels he or she can overcome, while the maladaptive strategies are responses to perceived threats that cause a larger amount of distress. Applying the Control-Value Theory of Achievement Emotions [2], one might gather that some students feel that a particular activity is important, but they do not feel like they have control over their performance or the activity in that particular classroom context, and therefore they feel threatened. The maladaptive coping strategy most commonly used to deal with an instructor asking for a volunteer to answer a question was to avoid eye contact/ not raise your hand. A prior study has shown that students self-report learning from instructor questioning even if they do not answer the question themselves [58], but assessments indicate that silent students learn less than their more vocal peers [59]. Students who coped with another strategy (positive self-talk before raising their hand) might have an academic advantage because they would either get their answer confirmed (boosting self-confidence) or have a misconception clarified by the instructor.

### Potential influences of student characteristics and instructor practices

Active learning engages students—but it often employs extrinsic motivation—and by forcing students to do activities, it reduces the students’s ability to choose what to do with their time in the classroom (challenge to autonomy), can reveal areas where they lack knowledge (challenge to competence), and can force them into uncomfortable social situations (challenge to relatedness). Each of these is an embedded part of Skinner’s coping categories. Self-determination theory indicates that meeting an individual’s needs for autonomy, competence, and relatedness sustains intrinsic motivation; it does not create intrinsic motivation where there was none [51]. This means that extrinsic motivation is still important to motivate students to learn about topics with which they are unfamiliar or uninterested. Autonomous types of extrinsic motivation, such as regulation through identification or introjected regulation, have been associated with engagement and optimal learning in academic contexts [60]. A model proposed by Ntoumanis et al [61]—based on social determination theory and cognitive-motivational-relational theory—indicated that stress appraisals, emotions, physiological response, and personality/disposition all influenced coping strategies. This theoretical underpinning explains why individual students are likely to have different coping strategies for dealing with different types of active learning, and why different students may cope with a single type of active learning differently.

Interestingly, of the four potential coping categories within the “responses to challenges or threats to autonomy” part of Skinner’s categorization, only one (accommodation) was found in the student response data set. Negotiation, submission, and opposition were never found independently by either of the data coders. Autonomy is the connection between one’s will and their actions, and reflects the extent to which the individual can exhibit the behaviors of their choice [32]. One potential explanation is that threats to autonomy are relatively lower in these classes than threats to competence or relatedness. Indeed, others have found that autonomy was not associated with academic achievement, even though it is associated with intrinsic goal orientation and self-efficacy [62]. One related factor may be the instructor and student power differential in an introductory classroom. Many of the students in this study were freshman in their first year of a majors’ Biology class so they may have been less likely to feel a threat to their autonomy or perhaps were unwilling to display overt push-back on classroom practices via the autonomy-related coping strategies. Student perceptions of autonomy also differ depending on whether they feel they had a choice in taking a course [63]. One suggestion for future studies would be to directly measure the types of threats that students perceive in order to try to relate that threat to their use of coping strategies.

Instructors can support student motivation, well-being, and positive emotions by supporting student competence, relatedness, and autonomy as they employ active learning in the classroom. Active learning itself is grounded in the principle of scaffolded practice, providing formative assessment that is designated to enhance student understanding [52], thus generating competence. Best practices for active learning suggest engaging students in peer conversations so that they can learn from the ideas of others [52], thus enhancing relatedness. Finally, intructors should strive to use autonomy-supportive practices that create open lines of communication about why they are using particular practices, as well as intentionally listening to student voice and feedback. When students perceive that their instructor uses autonomy-supportive practices, they report lower anxiety and increased perceived competence compared to their peers who perceive their instructor as using less autonomy-supportive practices [63]. Active learning practices that are explained by instructors and implemented in a supportive manner will be perceived by students differently than confusing or harsh practices [64], which will likely influence student anxiety levels and coping strategies. This is why implementation of active learning is so critical to its effectiveness [65].

The impact of differences in instructor implementation of active learning on student emotion and coping aligns with Pekrun’s theory [2], which predicts that emotions are sensitive to the learning environment as well as perceived control and value. So, for example, even though many instructors used clickers in their classes, there may be fundamental differences in how they used them in the classroom. Some instructors may have given points for correct answers on questions, while others may have only given participation points. Some instructors may have asked questions on readings students were supposed to do before class, while others may have probed student understanding of material that was just discussed in class. Moreover, of course, there were likely wide differences in the difficulty of questions that each instructor asked students. Each of these variations in implementation may have variable impacts on student emotional reactions.

Despite potential differences among instructor implementation in the classes examined in this study, we did detect similar patterns in how students were coping to different active learning practices across these multiple contexts. In addition, studies of anxiety across several institutions have identified uniform student reactions to certain active learning practices, such as cold calling inducing more anxiety than group work [25, 26]. Thus, although individual classes are expected to have variability compared to other classes, there may still be general trends not only in anxiety but also in coping to anxiety that apply to different active learning practices regardless of context.

### Limitations

There are several limitations to this study, including a student sample that did not represent the whole diversity of the population of students in the courses, a student sample with limited ethnic and gender diversity, and the use of a survey that was completed voluntarily and asked students to provide self-reports of their classroom experiences. For the survey, only a single item was used to assess student anxiety about each individual active learning technique. These items were not validated and may not necessarily be representative of the construct being measured. The survey did not measure the types of challenges or threats students perceived to their three psychological needs as described in Self-Determination Theory. The survey was administered to students at a single time-point in the semester, and we recognize that coping strategies might change over time or in the presence of different stressors outside the classroom. We also recognize that differences in instructor implementation of active learning might impact student anxiety and coping strategies, but we were not able to assess the details of implementation in each classroom. We were also not able to interview a sub-sample of students to more deeply probe their coping responses. However, this study provides a foundation upon which further studies delving into the causation of student anxiety and coping responses, functional impacts of coping strategies on student’s academic achievement, persistence, and emotional health, and possible remediation efforts to encourage the use of adaptive coping strategies may be designed.

### Implications

Student experiences of anxiety related to active learning practices, and the coping strategies they use to deal with these anxieties, are likely impacted by multiple factors inherent to students and their individual classroom environments. Coping strategies may influence health, well-being, behavior, emotion, and cognition [61], and therefore optimizing student coping to the anxieties they may feel in the active learning classroom may provide multiple benefits. Psychosocial interventions [66] may be one way to support student emotion regulation in response to anxiety in introductory Biology classrooms; such interventions were found to have positive impacts on persistence in one study [67]. Psychosocial interventions are brief activities performed in the classroom that aim to improve students’ thoughts, feelings, or beliefs in or about the classroom [66], and some have been shown to reduce anxiety in the classroom [68, 69]. One type of psychosocial intervention that could be employed is a self-affirmation intervention, which aims to reduce a person’s anxiety by reminding them of their many positive characteristics [66, 70]. Undergraduate students receiving a self-affirmation intervention had more self-affirming thoughts and fewer self-threatening thoughts when they were stressed than students who did not receive the intervention [68]. Attributional retraining interventions may also be helpful, in that they could guide students to redirect attribution of their academic performance from things that are out of their control (i.e. intrinsic level of intelligence) to things that are within their control (ie. level of effort) [71]. Given prior studies indicating a link between high general classroom anxiety and lack of persistence in the Biology major, we suggest that more work should be done to explore the frequency and effectiveness of coping strategies in introductory Biology classrooms as a way to support studies on emotion regulation and student success in Biology.

## Supporting information

S1 Table Coping strategy category descriptions and example student responses. (DOCX)

S1 Appendix Student survey data. (XLS)
