# Supplementary material for: How do undergraduates cope with anxiety resulting from active learning practices in introductory biology?
Source: PLoS One. 2020 Aug 12;15(8):e0236558. doi: 10.1371/journal.pone.0236558 (PMC7423088; doi:10.1371/journal.pone.0236558)
Supplement: S1 Table — (DOCX) [file pone.0236558.s001.docx]

**Table S1. Coping strategy category descriptions and example student responses**

| **Category and description** | **Example Student Responses and Boundary Cases** |
| --- | --- |
| Problem solving: The student adjusts their actions to be effective; they think, strategize, or plan. This may include practical actions such as having required materials readily available in class. | - I just break down the question in my head in order to answer the question with concepts we are going over in class that day. - I reread the question until it makes sense. - Think of an answer ahead of time - To reduce anxiety, I usually have MasteringBiology already pulled up, so I do not get anxious and feel like I am going to miss the clicker questions. - **Boundary:** Figure the question out. - **Boundary:** I try to provide the best reasoning that I have. |
| Information seeking: The student takes action to find additional contingencies. They obtain additional information by reading, observing, preparing, or asking others for help. | - Ask people around me to help - As long as I study and watch the Youtube [*sic*] lecture before class, the anxiety is decreased. - I try to look through my notes to find an answer to the question if I do not already know the answer. - Look for the smartest person around me - **Boundary:** Talk to others (in response to clicker question) - **Boundary:** When we talk about it with people around us I don't feel much anxiety (in response to clicker question) |
| Helplessness: The student finds the limits of their actions, and may expresses hopelessness, uncertainty, or panic. They may hope they don’t have to do the activity or freeze in fear. | - I hope to not get called on - I normally never know the answers - I begin to clench up and stare at the professor - The clicker questions are timed and over so quickly that it's difficult to use any coping mechanisms. - **Boundary:** I say the answer in a question tone and speak quietly. - **Boundary:** give it my best guess and hope i hit somewhere close |
| Escape: The student avoids the anxiety-causing activity or environment. They avoid acting or thinking. They may hide, avoid eye contact, distract themselves, or just not participate. | - Try to avoid answering the question - Try to avoid eye contact if I don't know the answer - I just try to slouch down in my seat so that the professor does not call on me since he can't see me in the back. - Keep quiet - **Boundary:** "I don't know" - **Boundary:** I try to always make sure that I'm not the one who has to answer the question |
| Self-reliance: The student protects their social resources and draws upon their own abilities. The student uses emotional or behavioral regulation. They engage in self-soothing or positive self-talk. | - Breathe - I try to feel confident in my answer. - I take a deep breath and remind myself that I want this. I want to be in the course, I want to ask stupid question and receive helpful criticism, and I want the ability to learn. - Chew gum - **Boundary:** I just stay calm. - **Boundary:** clear my throat and talk loudly |
| Support seeking: The student uses their available social resources. They reach out to others for comfort or emotional support (not information), or seek spiritual support. They rely on existing friends or creating new positive social interactions. | - Try to introduce myself and meet the others in the group. - Working with a group can be stressful but when you get started interacting that reduces my stress. - I try to sit with people I know so I know we can do good work together. - I pray. - **Boundary:** The anxiety comes with whether or not I can find a group to work with. The anxiety goes away once I find a group. - **Boundary:** Joke (in response to cold calling) |
| Delegation: The student finds the limit of their resources and lets other people do the work. This is a mal-adaptive variant on help-seeking. They may whine, complain, distract the group, or just wait for others to do the work. | - I don't insert myself or my opinion too much, I just sit on the fringe of the group and offer my input when asked. - Usually let other begin the conversation or answer first then add to or agree with what has been stated - Usually wait until someone else answers the question - I listen to others speak before I say anything - **Boundary:** I get nervous, so sometimes I just stay quiet.(in response to group work) - **Boundary:** I tell myself that someone else will answer. |
| Isolation: The student withdraws from a situation that they view as unsupportive. The student avoids social interaction. They work by themselves even when they are asked to work with others. | - If anything [*sic*] I will just work by myself - Not work with the class - I don't work with others unless the other person initiates. - I usually just don't work with others because there is no penalty - **Boundary:** No. I don't like to interact with people I don’t know. I also don’t like people who use me for answers so I keep to myself to reduce this "anxiety" - **Boundary:** I just don't do it. I'll work on my own. |
| Accommodation: The student flexibly adjusts their preferences to the available options. They do the required task, but may do it quickly or without much thought. | - I usually just answer it as fast as I can. - No. I just say what I think is the answer, and if i get it wrong, then I learn something new. - Try to answer the question as soon as possible - I typically don't enjoy group work but sometimes you've just got to deal with it. I don't really change my behavior just because I'm anxious. - **Boundary:** I try to work as fast as I can because there is a time limit and I most times do not have my answer by the end of that time. I get stressed from the time limit, not the question. - **Boundary:** I try to be as sure as possible about getting the right answer |
| Multiple categories | - I just do not raise my hand or volunteer myself rather than just waiting for someone else to answer and listen to the answer and the teachers discussion following (**Categories: Escape, Delegation**) - I usually don't answer, but if I have to, I just try to remember that everyone in there is just another student and trying to learn just like I am. It doesn't always work. **(Categories: Escape, Self-Reliance)** - I just evaluate the question and talk to my peers to see if we have the same answers. **(Problem solving, Information seeking)** - I try to take deep breaths and think through the question being asked, or ask the people around me. If it really starts to get to me, I just don't answer the question. **(Problem solving, Information seeking, Support seeking, Escape)** |
| No code | - I don't mind working with others. - Look at other people - Time constraints - I do not really mind answering class questions because it allows me to practice. |

These are examples of student responses to all nine coping categories that students reported, as well as responses that did not fit in any of the categories, typically because of ambiguity in the response (No code). Also included is a description of each category, as well as a tenth row showing an example of coding student responses into multiple categories—the categories agreed upon are in bold font at the end of each student response. Boundary cases (labeled as “Boundary”) are the student comments that we had the most difficulty coding; they represent what we determined to be the outer limits of what fits in a category.
